# The Effect of Exercise on the Prevention of Osteoporosis and Bone Angiogenesis

**DOI:** 10.1155/2019/8171897

**Published:** 2019-04-18

**Authors:** Xiaoyang Tong, Xi Chen, Shihua Zhang, Mei Huang, Xiaoyan Shen, Jiake Xu, Jun Zou

**Affiliations:** ^1^School of Kinesiology, Shanghai University of Sport, Shanghai, China; ^2^School of Sports Science, Wenzhou Medical University, Wenzhou, China; ^3^Department of Pharmacology, School of Pharmacy, Fudan University, Shanghai, China; ^4^School of Biomedical Sciences, University of Western Australia, Perth, Western Australia, Australia

## Abstract

Physical activity or appropriate exercise prevents the development of osteoporosis. However, the exact mechanism remains unclear although it is well accepted that exercise or mechanical loading regulates the hormones, cytokines, signaling pathways, and noncoding RNAs in bone. Accumulating evidence has shown that bone is a highly vascularized tissue, and dysregulation of vasculature is associated with many bone diseases such as osteoporosis or osteoarthritis. In addition, exercise or mechanical loading regulates bone vascularization in bone microenvironment via the modulation of angiogenic mediators, which play a crucial role in maintaining skeletal health. This review discusses the effects of exercise and its underlying mechanisms for osteoporosis prevention, as well as an angiogenic and osteogenic coupling in response to exercise.

## 1. Introduction

Osteoporosis is a skeletal disease characterized by low bone mass or bone mineral density (BMD), deterioration of bone micro-architecture, and increased risk of fracture [1, 2]. The worldwide rapid growth of the aging population has been implicated in many aspects of human health, and among these osteoporosis has been one of the main public health problems in aging people, particularly for individuals aged above 50 years [3, 4]. Previous studies have shown that exercise or physical training might improve bone mass and strength and consequently promote bone formation, which could effectively treat and prevent osteoporosis with no side effects, in preference to treatments that rely excessively on pharmacological intervention by the use of antiosteoporosis drugs [5–8]. Thus, exercise has been recommended by WHO as physical therapy for non-drug osteoporosis prevention and treatment [9, 10]. Although the exact mechanisms of the beneficial effects of exercise on skeletal health are far from being fully understood, it is well known that the changes mediated by exercise are beneficial for bone health from many different aspects, such as force stimuli, hormones, cytokines, and cell signaling pathways, as well as noncoding RNAs.

## 2. The Potential Mechanisms of Exercise in the Prevention of Osteoporosis

### 2.1. Mechanical Loadings Induced by Exercise Promote Bone Formation

Studies have established that exercise and sports activities can render mechanical stimuli to the joint tissues and bone which are needed to maintain the tissue properties [11]. The dynamic balance between bone formation and resorption maintains adult skeletal health. Mechanical loadings including compression, strain, and fluid shear are the stimuli that play essential roles in osteoblast differentiation and mineralization, as well as maintaining the proper high bone mass and density [12, 13]. On the contrary, unloading causes the loss of human bone mass and even osteoporosis. Studies of astronauts living in the space revealed that the astronauts lost bone mass at the speed of 2% per month in the hip [14]. A report by Lloyd et al. also showed that a weightlessness environment caused the loss of bone mass in animals as unloading mice developed lower bone mass than the control group [15].

Exercise or physical activities produce multiple mechanical loadings, such as tension, compressive, and fluid shear stress, which have beneficial effects on reducing bone loss, increasing bone strength, and preventing osteoporosis in aging people. Studies have shown that the increased forces impacting the body during exercise are correlated with elevated bone mass density and bone strength in athletes [16]. Further, a systematic review and meta-analysis has concluded that exercise appeared to positively influence osteogenesis and skeletal geometry in a force regional manner [17].

### 2.2. Hormones and Cytokines Induced by Exercise Promote Bone Formation

Exercise regulates hormones in the body such as estrogen, parathyroid hormone, and glucocorticoids, which may be another key mechanism in bone metabolism and remodeling [18–21]. Bentz et al. reported that physical activity could promote the secretion of estrogen (estradiol) in premenopausal women and partially mimic the effects of hormone replacement treatment for osteoporosis [22]. Similarly, exercise could increase serum estradiol (E2) level, which is consistent with the increase of bone mass and strength in ovariectomized rats [23, 24]. Further, resistance training also showed that exercise could increase serum testosterone levels in elderly men, which was accompanied by reduced bone loss [25].

Hormones not only directly regulate bone metabolism, but also induce or coordinate cytokines in the regulation of bone metabolism. For instance, estrogen can inhibit bone resorption by inhibiting TRPV5 and RANKL expression and promoting OPG expression. In addition, cytokines also play key roles in the balance of bone formation and resorption. Santos et al. reported that six-month moderate exercise could reduce bone loss in the elderly, which appears to be the result of an increased serum level of IL-10, while significantly reducing the serum levels of IL-6, and TNF-*α* [26]. Further, exercise has been shown to decrease the secretion of proinflammatory cytokines of bone resorption, such as IL-1, IL-6, and TNF-*α*, and to increase the protective cytokines against bone resorption, such as IL-2, IL-10, IL-12, IL-13, IL-18, and IFN [27].

### 2.3. Signaling Pathways Induced by Exercise Promotes Bone Formation

Signaling pathways such as Wnt/*β*-catenin, BMP, OPG/RANKL/RANK, and Notch play a leading role in regulating bone metabolism [28]. Previous studies demonstrate that exercise or physical training could promote osteoblast differentiation, inhibit osteoclast activity, and improve bone remodeling through the regulation of multiple signaling pathways, such as upregulating Wnt/*β*-catenin, BMP, and OPG/RANKL/RANK signal pathways, resulting in improved bone formation and the prevention of osteoporosis [27, 29–33]. Additional pathways activated by mechanical stress or exercise may involve PERK-eIF2*α*-ATF4, mTORC2-Akt-GSK3*β*, or PI3K/Akt/GSK-3*β*/*β*-catenin, which are also important in the positive regulation of bone metabolism [34–36].

### 2.4. Noncoding RNA Induced by Exercise Promotes Bone Formation

Recent studies have found that noncoding RNAs, including siRNA, microRNAs, lncRNA, and circRNA, are widely involved in the regulation of various stages of bone metabolism, including the proliferation and differentiation of osteoblasts and osteoclasts [28, 29].* In vitro*, findings indicate that mechanical stress stimulates the expression of microRNAs, which might serve as potential therapeutic candidates for the prevention and treatment of bone diseases, particularly for osteoporosis [30–32]. For instance, Guo et al. found that mechanical distraction upregulated the expression of miRNA-191 and miRNA-3070a and downregulated the expression of miRNA-218 and miRNA-33 and that the target genes of these differentially expressed miRNAs were involved in regulating osteoblastic differentiation [33]. Further,* in vitro* and* in vivo* findings appear to have discovered that mechanical loading decreases the expression of miRNA-103a and its host gene, PANK3, and increases the expression of* Runx2* (the master transcription factor of osteogenesis), indicating that the downregulation of miRNA-103a may be a key mechanism for the mechanical stimulation of bone formation [34]. Recently, long noncoding RNAs (lncRNAs) have been explored for their roles in the regulation of bone metabolism [35, 36]. More studies will be required to determine whether long noncoding RNAs are involved in the prevention of osteoporosis mediated by physical therapy.

## 3. Bone Angiogenesis and Osteoporosis

Bone is a highly vascularized tissue with a wide network of blood vessels and capillaries that provide oxygen and nutrients for bone formation and development, which are mediated via the regulation of different signaling pathways between endothelial cells and bone cells [37, 38]. Blood vessels also play essential roles in the process of osteoporosis and are formed via two distinct biological processes. In the early stages of embryogenesis, hemangioblasts are derived from mesodermal cells, which migrate to a specific site and aggregate to form the primary vessels in the process of vasculogenesis [39]. Subsequently, most of the new blood vessels sprout by the process of angiogenesis, which is accompanied by an expansion of the existing vascular networks through multiple steps such as endothelial cell proliferation, migration, sprouting vessel pruning, and anastomosis [40, 41]. Thus, it appears that the vasculature in bone is formed mainly by angiogenesis.

### 3.1. Bone Angiogenesis Is a Potential Target for the Prevention of Osteoporosis

Bone formation occurs in two different ways: one is endochondral ossification and the other is intramembranous ossification. Endochondral bone formation requires the provision of bone-forming osteoblasts, and progressive neovascularization accompanied with growing bone. Overall, bone formation occurs in a spatial and temporal relationship with vascularization of the ossifying tissue, which is called angiogenesis-osteogenesis coupling [42–44]. In the process of angiogenesis, endothelial cells proliferate, migrate, form tubes, and eventually produce conduits where blood flows and provides the necessary nutrients, oxygen, growth factors, and hormones for the bone cells. Additionally, hematopoietic precursors of osteoclasts are also transmitted by blood vessels to the sites of cartilage and bone resorption in order to eliminate the end-products of the degraded extracellular matrix. Moreover, the subendothelial walls of vessels consist of pericytes, which appear to be an important cell involved with the coupling between osteogenesis and angiogenesis [45, 46].

Angiogenesis in the bone microenvironment is required for bone growth and development, postfracture repair, and maintenance of normal bone health [47, 48]. For example, Vogt and Alagiakrishnan found that the blood supply in the people with osteoporosis or osteopenia is relatively lower than that in the people with normal bone mass, indicating that bone blood supply and bone mineral density are highly correlated [49, 50]. Further, Ramasamy et al. found that endothelial Notch signaling promotes angiogenesis and osteogenesis in the bone microenvironment, which was evidenced by the presence of 5-ethynyl-2′-deoxyuridine (EdU) labeled vascular endothelial cells in the area where long bones grew vigorously in mice [51]. Additionally, Notch signaling in endothelial cells appears to be involved with the age-dependent regulation of hematopoietic stem cell niches in bone, which is accompanied by increases in the number of CD31-positive capillaries, platelet-derived growth factor receptor-*β* (PDGFR*β*)-positive perivascular cells, and arteriole formation [52]. Therefore, activating angiogenesis in the bone microenvironment might be an important strategy in the prevention of osteoporosis.

### 3.2. The Bone Angiogenesis Induced by Exercise

Previous studies have shown that exercise-mechanical loading stimulated angiogenic-osteogenic responses in bone. For example, Matsuzaki et al. showed that skeletal fatigue loading leads to an increase in periosteal vascularity and regional bone area and that angiogenesis-osteogenesis is spatially coordinated in response to different mechanical stimulation [53]. Increases in regional marrow and bone blood flow during exercise may also reveal that the dynamic generation of hydrostatic pressure gradients between bone capillary efferent and the medullary cavity could promote bone interstitial fluid flow [54]. In addition, the mechanical loading provides compressive forces for distinct regions of bone, and bone interstitial fluid can flow from high fluid pressure area to the low one [55], which stimulates osteoblastic osteogenesis and decreases osteoclast formation [56]. Further, Stabley et al. also showed an increase in marrow blood flow and generalized hind limb bone during physical activity following exercise training for some time [57]. Yao et al. found that only two weeks of running exercise could increase vessel number in the proximal metaphysis in rats, and significant changes of BMD in response to exercise occurred five weeks after training [58]. Boerckel et al. also found that delayed mechanical loading enhanced bone formation by stimulating vascular remodeling in a rat model, while early mechanical loading inhibited this process, suggesting that vascular networks and its coupling effect on bone regeneration occur in a time-dependent manner in response to mechanical loading [59].

## 4. Factors Induced by Exercise Regulate Bone Angiogenesis

Many factors, such as VEGF, HIF-1, EGFL, NPNT, and Notch ligands, regulate the proliferation and differentiation of endothelial cells and promote bone vascularization, as well as an angiogenic and osteogenic coupling in the bone local environment [60, 61]. Here, we provide an update regarding the effects of exercise or mechanical loading on these factors.

### 4.1. VEGF

Vascular endothelial growth factor (VEGF) belongs to the dimeric protein family, including six members: VEGF(-A, -B, -C, -D, -E) and PlGF (placental growth factor) [62]. VEGFs are abundantly expressed and play an important role in the proliferation, migration, and activation of endothelial cells, as well as in angiogenesis [63, 64]. The receptors of VEGFs include VEGFR(-1, -2 -3), Npr1, and Npr2 [65]. Among them, the expression of VEGFR1 is mainly in hematopoietic stem cells, the expression of VEGFR2 in vascular endothelial cells, and the expression of VEGFR3 in lymphocyte-endothelial cells [64]. High level of expression of VEGF was found in the mineralized regions with low hypertrophy of the cartilage of embryonic bones, and increased angiogenesis in these areas correlates with ossification [66]. In addition, the expression of VEGF increased during osteoblast differentiation, which could promote angiogenesis, bone formation, and remodeling [67, 68]. Further, VEGF/VEGFR2 signaling also regulates osteogenic-related factors, such as *β*-catenin and Notch2 [69]. In summary, VEGF promotes angiogenesis in bone, which is beneficial to “angiogenesis-osteogenesis” coupling, bone formation, and remodeling.

A number of studies have revealed that exercise including running and resistance exercise increases the expression of VEGF in the brain, lung, and skeletal muscle [70, 71]. Two weeks of running exercise appears to increase vessel number in rats. Running exercise also upregulated the expression of VEGF and VEGFR1 in periosteum and metaphyseal bone [58]. However, these effects of exercise on angiogenesis were inhibited when VEGF blockade was used in the five-week training group. These findings indicate that angiogenesis mediated by VEGF is one of the main factors in exercise-induced bone formation [58]. Other studies also showed that the expression of VEGF and angiogenesis in the growth plate was affected by mechanical signals [72, 73]. Liu et al. revealed that mechanical loading had a cumulative effect on the expression of VEGF mRNA, which upregulated bone remodeling signals in osteocytes at the early time point [74]. Similarly, Groothuis et al. found that appropriate mechanical stimuli enhanced VEGF and endothelial tube formation, and this proangiogenic effect was suppressed by inhibition of VEGFR2 signaling [75]. Collectively, these studies indicate that exercise or mechanical loading can increase angiogenesis in bone through the regulation of VEGF expression and function.

### 4.2. HIF

Hypoxia activates a variety of intracellular signaling pathways and regulates target genes by the transcriptional factor, HIF (hypoxia-inducible factor). One of the three *α*- subunits [HIF1*α*, -2*α*, and -3*α*] and *β*-subunit (HIF1*β*, or ARNT) form HIF heterodimer [76]. Many biochemical processes involve HIF target genes, such as anaerobic metabolism and angiogenesis. Wang et al. showed that activation of the HIF1*α* pathway increased blood vessel formation in bone and was involved in bone remodeling [77]. Knockout of the HIF1*α* gene in osteoblasts resulted in decreased bone vascularization and osteogenesis. Conversely, activation of the HIF signaling pathway in osteoblasts not only inhibits bone loss caused by estrogen deficiency but also promotes bone formation and angiogenesis [78]. Moreover, HIF-1 regulates its downstream target gene—VEGF, and the combination of these factors plays important roles in vascular-bone coupling [79].

Previous studies have demonstrated that exercise or physical activity could increase the level of HIF in skeletal muscle, which is involved in the angiogenesis process [80–82]. Ribeiro et al. showed that a bout of resistance exercise promotes the increases in EPCs and the expression of angiogenic genes such as VEGF and HIF1*α* [80]. Further, Rodriguez et al. found that eccentric exercise stimulates a HIF1*α* response through the upregulation of eNOS and VEGF gene expression in untrained skeletal muscle [81]. It is likely that the adaptation to endurance training may increase the effectiveness of HIF1*α* on angiogenesis, which improves tissue function during low oxygen conditions. Other studies also indicated that mechanical loading activated the expression of HIF1*α* gene. For instance, nondamaging mechanical loading could improve the bone formation in mice, but this process was suppressed in HIF1*α*Δ mice, suggesting that the response of angiogenesis to mechanical loading is mediated via HIF1*α* signaling [83]. Another study also found that the release and expression of ANGPTL4 protein could be stimulated by cyclic stretching of human tendon fibroblasts via HIF-1*α* signaling, leading to a proangiogenic effect [84]. Taken together, these studies all supported that HIF-1*α* is a factor stimulated by exercise or physical activity, potentially involved in the bone angiogenesis, bone formation, and remodeling.

### 4.3. FGF

Fibroblast growth factors (FGFs) belong to a family of eighteen different ligands and play important functions in cell survival, proliferation, and differentiation through four different tyrosine kinase receptors (FGFR-1, -2, -3, and -4) [85]. Studies have shown that FGFs display a key regulatory role in bone angiogenesis [86]. One study revealed that the expression of the tight junction protein ZO-1 (or TJP1) and the vascular endothelial adhesion molecule VE-cadherin (or cadherin 5) was increased by systemic injection of FGF2 or basic FGF, accompanied with the arterial vasculature expanded in bone [87]. Further, Kigami et al. revealed that FGF2 increased angiogenesis and promoted bone formation in rat calvarial critical-sized bone defects [88]. In addition, the loss of perivascular cells and increased vessel permeability associated with bone vessels were evident by inactivation of genes encoding FGFR1/2 in endothelial cells, suggesting that FGFR1/2 signaling maintains vascular integrity and arterial function in bone [87]. Collectively, these studies indicated that the FGF signaling pathways play an important role in the regulation of bone angiogenesis and osteogenesis.

Previous studies showed that exercise leads to increased FGFs expression. For instance, one study demonstrated that a period of voluntary wheel-running enhanced the expression of FGF-2 in the brain [89]. Andrzejewski et al. showed that ten weeks of running exercise increased the mRNA expression of FGF-2 and VEGF in rat tendon [90]. There is good evidence that FGF-2 is increased in muscle injury induced by exercise, potentially resulting in enhanced angiogenesis and osteogenic effects. Moreover, mechanical loading such as cyclic tension strain increased FGF2 protein expression in human periodontal ligament cells [91]. In all, these studies indicated that exercise-induced FGFs could regulate bone angiogenesis and serve as potential candidates for the prevention and treatment of osteoporosis.

### 4.4. MMP

MMPs (matrix metalloproteinases) are mainly secreted by osteoclasts and vascular cells, and accumulating evidence suggests that MMPs take an active part in bone angiogenesis and bone remodeling, particularly MMP-2, -9, and -13. Stickens et al. found that MMP13 mutant mice exhibited an increase in growth plate and flat bone. In contrast, mice with both MMP13 and MMP9 deletions showed a decrease in intrachondral angiogenesis and ECM remodeling [92]. In addition, Cackowski et al. also showed that MMP-9 regulates angiogenesis mainly by affecting the migration of osteoclasts [93].

Many studies investigating the regulation of MMPs by exercise were conducted in skeletal muscle. In addition, Ross et al. investigated the endothelial progenitor cell factors in the circulation after endurance resistance exercise in trained men and found that exercise enhanced VEGFs (VEGF-A, VEGF-C, and VEGF-D) and MMPs (MMP-1, -2, -3, and -9) in serum. It is believed that the change of MMPs induced by exercise would be beneficial to bone angiogenesis and bone formation. Similarly, other studies revealed that mechanical loading stimulated MMPs such as MMP-9 and -13, which also play essential roles in bone angiogenesis [94, 95].

### 4.5. Notch

There is no doubt that Notch plays an important role in bone metabolism and bone angiogenesis [96, 97]. The proangiogenic effect of VEGF signaling is modulated by Notch signaling in ECs. Remarkably, activation of Notch signaling in bone was found to stimulate local osteogenesis and angiogenesis, as well as the formation of VEGF-producing chondrocytes in the adjacent growth plate [51]. In addition, Notch-1, Notch-3, and Jagged-1 also play a key role in bone angiogenesis [98]. Many studies have reported that exercise or mechanical loading stimulated the Notch signaling pathway [99–101]; however, the effects of exercise on the regulation of Notch and angiogenesis in bone, and on the prevention of osteoporosis, require further investigation.

**Figure 1:**
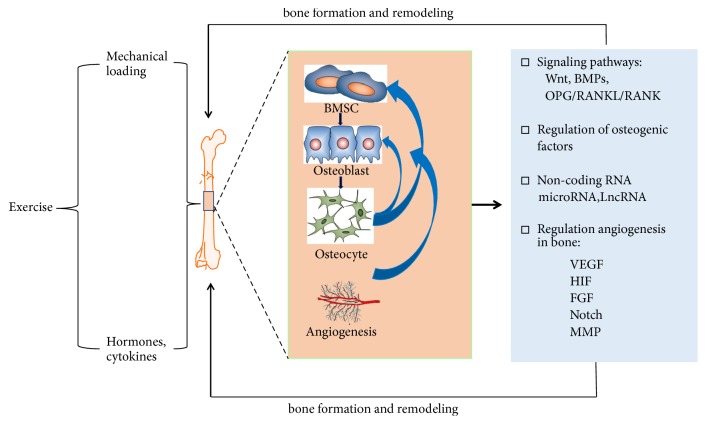
The potential mechanisms of exercise in promoting osteogenesis and angiogenesis.

Putting together, angiogenic regulators such as VEGF, HIF, FGF, and their signaling pathways were necessary for the regulation of angiogenesis [102], which was beneficial for bone formation and osteogenesis. Interestingly, these angiogenic mediators can be activated by exercise or mechanical loading (Figure 1). Although most studies examining the effects of exercise on these angiogenesis were conducted on skeletal muscles, the effects of exercise training or mechanical loading on the prevention of osteoporosis via angiogenesis partially as the interface of bone and muscle are highly possible. However, more studies are still needed to investigate the effects of exercise on the regulation of angiogenesis and osteogenesis coupling.

## 5. Conclusion

Exercise or physical training can prevent osteoporosis in the elderly as a non-drug preventive strategy. The interaction of mechanical loading, hormones or cytokines, and signaling pathways induced by exercise increased bone formation and reduced bone resorption, leading to the maintenance of healthy skeleton. Dysregulation of bone angiogenesis is associated with many bone diseases including osteoporosis, and exercise improves angiogenesis in bone via the regulation of key angiogenic mediators. Further understanding the mechanisms of angiogenesis, signaling pathways, and key regulators induced by exercise will lay the foundation for the prevention of osteoporosis in the aging population.
